# The effects of war on digital adherence technology engagement for TB treatment in Ukraine

**DOI:** 10.5588/ijtldopen.25.0503

**Published:** 2026-01-09

**Authors:** N. Deyanova, C.F. McQuaid, V. Kochanov, A. Bogdanov, N. Madden, S. Charalambous, K. van Kalmthout, D. Jerene, K.L. Fielding, K. Gamazina

**Affiliations:** 1 Organization for Appropriate Technologies in Health, Kyiv, Ukraine;; 2 TB Centre, Department of Infectious Disease Epidemiology, LSHTM, London, UK;; 3 Program for Appropriate Technology in Health, Kyiv, Ukraine;; 4 KNCV Tuberculosefonds, The Hague, the Netherlands;; 5 School of Public Health, University of the Witwatersrand, Johannesburg, South Africa;; 6 Aurum Institute, Johannesburg, South Africa.

**Keywords:** tuberculosis, DAT, migration, armed conflict and TB care

## Abstract

**BACKGROUND:**

The Russian invasion of Ukraine in February 2022 has disrupted TB care. We examine the effects of the war on people with drug-susceptible TB (PWTB) and health care worker (HCW) engagement with a digital adherence technology (DAT).

**METHODS:**

We conducted a cluster-randomised trial of a DAT intervention in Ukraine. In the intervention arm, PWTB received a pillbox with a daily treatment reminder. Pillbox openings were captured real-time onto a platform, accessed by HCWs who could add manual doses where relevant. We compared DAT engagement in pre-, early-, and later-war periods.

**RESULTS:**

From June 2021 to 2022, 816 PWTB (32% women, median age 44 years) were enrolled. DAT engagement varied across regions and time period, with a decline in engagement post-February 2022. PWTB DAT engagement was 78%–84% of treatment-days. There was a three- to four-fold increase in unknown dose or delays in manual reporting of adherence (>7 days) post-February 2022 versus pre-war in Mykolaivska and Donetska oblasts and a two-fold increase in Odeska, Lvivska, and Zakarpatska oblasts.

**CONCLUSION:**

The war significantly disrupted DAT engagement, particularly in regions heavily affected by the conflict. Reduced engagement was likely due to migration and communication challenges. There is a critical need for resilient TB care in conflict settings.

The harmful effects of war on infectious diseases in civilian populations have been described.^1–3^ However, a deeper understanding of the mechanisms underlying these effects is rarely explored.^4,5^ The impact of armed conflicts on TB control programmes is a particularly relevant case. Across countries, there is a striking variation in the impact of armed conflicts on TB notifications, in contrast to uniformly satisfactory outcomes of most TB programmes and interventions conducted in complex emergencies.^6–8^ Most research has been limited to TB notifications and treatment outcomes in illustrating the extent of war on TB care, without any further in-depth understanding of patterns observed.^7^

The invasion of Ukraine by Russia on 24 February 2022 disrupted health service delivery,^9–11^ including for TB care.^12^ There was an increase in patients receiving care in a region away from their permanent residence due to internal displacement of the population and shortages of drugs and consumables for drug susceptibility testing.^12,13^ As a result, people with TB may be less likely to be able to access life-saving treatment or support to help them complete a treatment course.^2^ Among the evacuated civilian population were health care personnel, being forced^14^ to leave their homes and workplaces, which also caused a lack of health care providers in places.^9^ The Russian invasion began in the middle of the 18-month research phase of a cluster-randomised trial (CRT) of digital adherence technologies (DATs), conducted by the Adherence Support Coalition to End TB (ASCENT) consortium.^15^ The study was part of a pragmatic CRT in South Africa, Tanzania, The Philippines, and Ukraine, to evaluate whether DATs can improve the treatment success rate among adults with drug-susceptible (DS) TB. DATs such as smart pillboxes have been proposed as an alternative to delivering TB treatment, including directly observed therapy (DOT). In real-time, health care workers (HCWs) can access data on a patient’s engagement with the DAT, as a proxy for treatment adherence, without requiring patients to travel to the health care facility. However, data are limited, as to whether these interventions can improve treatment outcomes.^16,17^

In Ukraine, the DAT intervention being assessed in the CRT is a smart pillbox that records a patient’s daily box opening in real-time by sending a signal to an adherence platform. The HCW can monitor DAT engagement and respond when the box is not opened (a proxy for a missed dose), in a timely way by reaching out to the patients remotely and offering counselling and psychosocial support. This patient–HCW interaction, inherent in many DAT interventions, may be crucial in changing patient’s adherence patterns, offering an intermediate step in TB treatment delivery. This study aimed to assess whether the HCW’s interaction with the adherence platform data on patient engagement with the pillbox has changed over the war periods and geographical areas, in the intervention arm of the ASCENT trial in Ukraine.

## METHODS

### Parent study

As a part of the ASCENT project on digital treatment adherence support, we conducted a pragmatic cluster-randomised trial in 24 rayons from five oblasts in Ukraine.^15^ Facilities were randomised, in a ratio of 1:1, to standard of care or the DAT intervention based on a smart pillbox with differentiated care for those with adherence issues, triggered by the patient not opening the pillbox. In intervention facilities, adults aged ≥ 18 years with DS-TB starting treatment were given the pillbox, following written informed consent. Participants’ engagement with the pillbox was recorded in real-time onto a digital platform, which was accessed by HCWs. Pillboxes contained sufficient medication until the patient was scheduled to return to the facility for a monthly meeting with the HCW. Pillbox recharging occurred every 2–3 months, in most cases by the HCW, although some patients preferred to charge the module themselves at home. The 12 rayons randomised to the intervention arm represent 13 specialised TB facilities that provide the patient population with TB services and were situated in eastern (Donetska), southern (Mykolaivska and Odeska oblasts), and western (Lvivska and Zakarpatska oblasts) Ukraine. Facilities implementing the intervention include ambulatory departments of regional-level TB medical centres, TB rooms in multi-profile rayons and city hospitals, and TB rooms in TB dispensaries.

Following the start of the war with Russia in February 2022, the feasibility of delivering the trial intervention had been varied: in Mykolaivska and Donetska oblasts, many HCWs and trial participants had left the area to safer regions of Ukraine or abroad; in Odeska, Lvivska, and Zakarpatska oblasts, a few HCWs left their work, though some study participants temporarily left these regions during the first month of the war, and then returned to their homes (Supplementary Data Figure S1). Soon after the invasion and for most of the time during the war, however, the internet has been available in the whole country besides periods when power was off, though these periods lasted no more than several days. The mobile telephone connection was also available with the same limitation.

### Current study

We analysed engagement rates of patients and HCWs with the smart pillbox, using data recorded on the digital platform in the intervention arm clusters. Analysis was restricted to TB patients started on the pillbox from the 15 June 2021 to 30 June 2022 (main enrolment period for the trial), and using treatment-day follow-up through to 28 October 2022. The platform recorded for each patient a treatment-day as either: digitally recorded (pillbox opened on the day); manually recorded (no recording that the pillbox was opened and the HCW has confirmed a dose was taken through a phone call with the patient, potentially as a result of poor pillbox connectivity or charge, or because additional doses had been removed from the pillbox previously and taken on this date); dose missed (no recording that the pillbox was opened and the HCW has confirmed a dose was not taken by phoning the patient); and unknown (no recording that the pillbox was opened and no additional information from the HCW). HCW’s confirmation that a dose was or was not taken could take place at any time on or after the treatment-day. We define a treatment-day with no information as a combination of unknown or a manual dose added >7 days after the treatment-day. This definition reflects poor engagement with the DAT potentially from a patient perspective, as the pillbox was not opened in a timely way, and from an HCW perspective, as their engagement with the DATs intervention on a treatment-day was unknown or it was delayed by more than 7 days. For treatment-days recorded as unknown, the system does not capture whether the HCW had made an unsuccessful contact with the patient when the pillbox was not opened.

We grouped treatment-days in three time intervals: pre-war period (before 24 February 2022), the early phase of the war (24 February 2022–30 April 2022), and the later phase of the war (1 May 2022–28 October 2022). We separated rayons into predefined war-affected oblasts (Mykolaivska, Donetska) and those less affected by the war in the South (Odeska oblast) and West (Lvivska and Zakarpatska oblasts). Data were collapsed to a week and patient-level and the count of no information treatment-days per week was analysed using the negative binomial regression model with a random effect for cluster. We assessed whether the effect of time period on outcome differed by geographical area, by fitting an interaction between the variables, adjusting for age, sex, and treatment phase (intensive or continuation).

### Ethical statement

The trial was approved by the Ethics Commission of Public Health Center of the MOH of Ukraine (Ukraine IRB 2019-33), PATH Research Ethics Committee, the London School of Hygiene and Tropical Medicine Ethics Committee, and the WHO Ethics Review Committee.

## RESULTS

**Figure 1.**
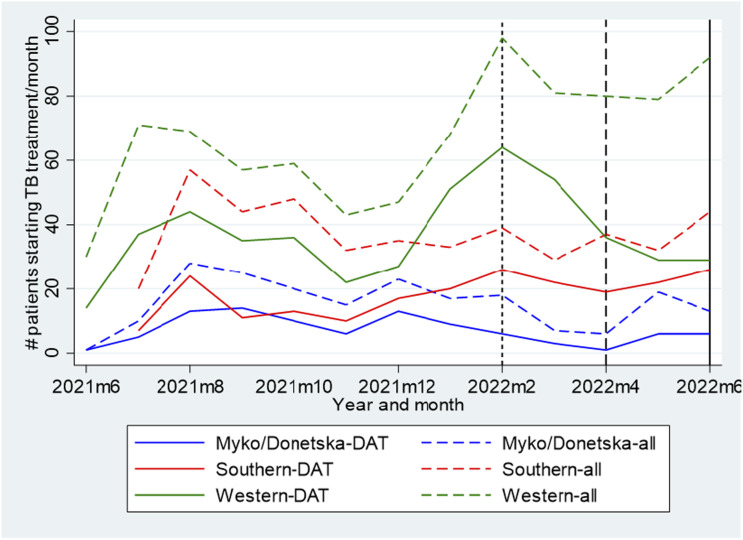
The number of all TB registrations and DAT registrations per month, by area (June 2021–June 2022). Vertical dotted line represents start of Russian invasion (late February 2022); vertical dashed line represents end of April 2022; vertical solid line represents the end of the enrolment period (June 2022). Region-specific lines represent total number of people starting TB treatment per month (dashed lines) and total number of people starting a pillbox for TB treatment per month (solid lines).

From 14 June 2021 to 27 June 2022, a total of 816 adults with DS-TB started the pillbox from 12 rayons (32% women, median age was 44 years, interquartile range: 23–54). The treatment initiation rate increased slightly over the three time periods, whereas the DAT initiation rate fell from 67.3/month pre-war to 25.7/month >4 months after the start of the war (Table 1, Figure 1). Over the entire enrolment period, DATs coverage (of all adult DS-TB registrations) was 54% in Mykolaivska and Donetska oblasts, 47% in Odeska oblast, and 57% in Lvivska and Zakarpatska oblasts (Table 1).

**Table 1.** Summary of study participants starting on the DATs over the analysis period and rate of starting DATs per month, and the treatment initiation rate per month, overall and by geographical area.

	Mykolaivska/Donetska	Odeska (southern)	Lvivska/Zakarpatska (western)	Overall
Number of rayons	3	3	6	12
Numbers starting on DAT/all individuals starting TB treatment *n*/*N*, %	109/202 (54.0%)	212/450 (47.1%)	495/874 (56.6%)	816/1,526 (53.5%)
Sex: *n* (%)				
Female	41 (37.6%)	63 (29.7%)	159 (32.1%)	263 (32.2%)
Male	68 (62.4%)	149 (70.3%)	336 (67.9%)	553 (67.8%)
Males, by time period				
Before war	61/99 (61.6%)	103/148 (69.6%)	251/371 (67.7%)	415/618 (67.2%)
26 February–29 April 2022	3/5 (60.0%)	32/42 (76.2%)	64/94 (68.1%)	99/141 (70.2%)
30 April–30 June 2022	4/5 (80.0%)	14/22 (63.6%)	21/30 (70.0%)	39/57 (68.4%)
Age, years				
Median (IQR)^**A**^	42 (36–51)	42 (34–49)	46 (36–58)	44 (23–54)
Starting DATs/month^**B**^				
Time period	Participants starting on DAT (rate/month)	Participants starting on DAT (rate/month)	Participants starting on DAT (rate/month)	Participants starting on DAT (rate/month)
Before war	99 (10.8)	148 (16.1)	371 (40.4)	618 (67.3)
26 February–29 April 2022	5 (2.2)	42 (18.7)	94 (41.8)	141 (62.7)
30 April–30 June 2022	5 (2.3)	22 (9.9)	30 (13.5)	57 (25.7)
Starting treatment/month^**B**^				
Time period	Adults starting treatment (rate/month)	Adults starting treatment (rate/month)	Adults starting treatment (rate/month)	Adults starting treatment (rate/month)
Before war	157 (13.9)	307 (27.1)	541 (47.8)	1,005 (88.8)
26 February–29 April 2022	13 (5.8)	67 (29.8)	161 (71.6)	241 (107.1)
30 April–30 June 2022	32 (14.5)	76 (34.3)	172 (77.7)	280 (126.1)

IQR = interquartile range.

A Age missing for *n* = 3 participants (*n* = 1 in Mykolaivska/Donetska and *n* = 2 in Lvivska/Zakarpatska oblasts).

B Month considered as 28 days.

**Figure 2.**
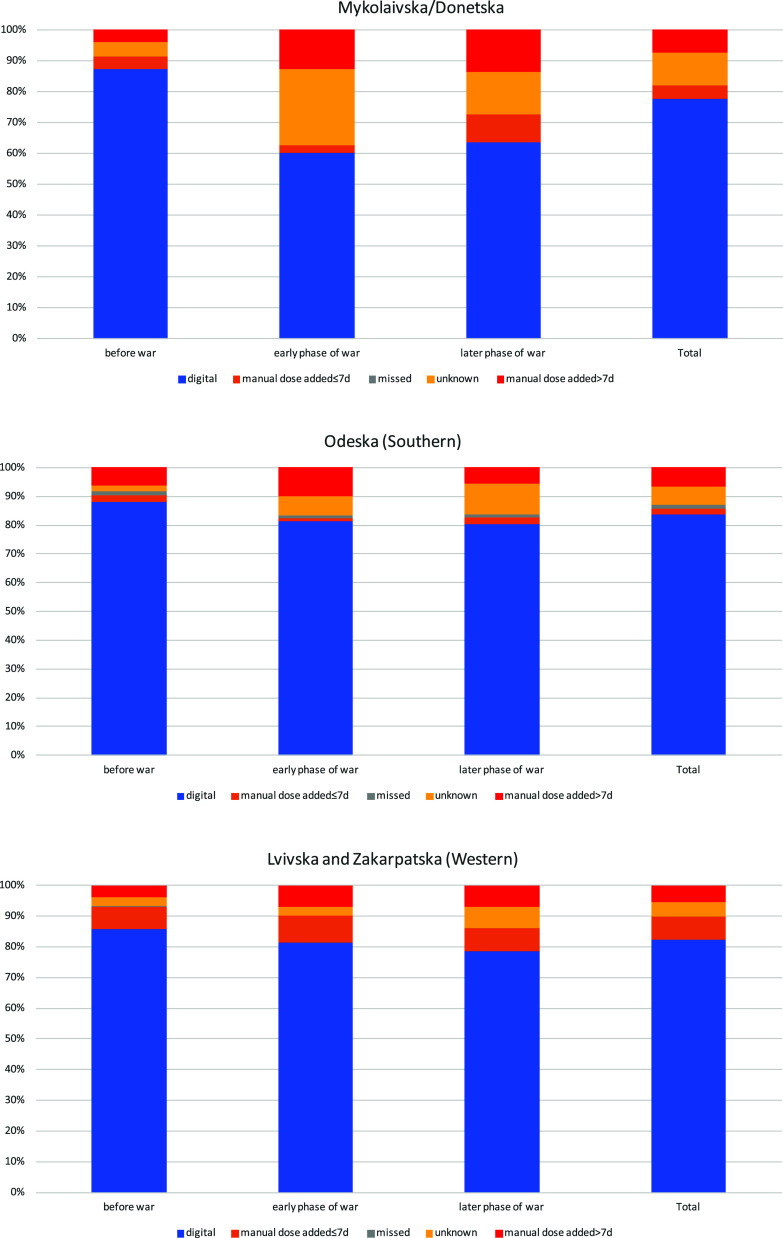
Distribution of smart pillbox engagement overall and by the three time periods, stratified by geographical area. Digital = box opened on the treatment-day; manual dose added ≤7 days = health care worker (HCW) added a dose ≤7 days of the treatment-day; missed = HCW has confirmed that a dose was not taken on the treatment-day; unknown = box not opened and no information from HCW; manual doses added >7 days = HCW added a dose >7 days of the treatment-day. Smart pillbox engagement is combined across participants on treatment in each time period.

Type of engagement differed by region and time period (Figure 2). In Mykolaivska and Donetska oblasts, the early phase of the war saw 25% of treatment-days with unknown engagement, which reduced to 14% in the later phase. Manual dosing added more than 7 days after the treatment-day was also common post start of the war (14%). In the Odeska oblast (southern region), manual dosing added within 7 days was uncommon (2%), whereas manual dosing added >7 days after the treatment-day accounted for 7% of treatment-days overall. The percentage of treatment-days with unknown engagement increased from 2% pre-war to 11% >4 months after the start of the war. In Lvivska and Zakarpatska oblasts (western region), manual dosing added within 7 days and greater than 7 days was 8% and 6%, respectively. The percentage of treatment-days with unknown engagement was 3% in the first two time periods and 7% in the later phase of the war.

The percentage of treatment-days per week where there was no information (unknown or manual dose added >7 days after treatment-day) for DAT engagement increased following the start of the war, particularly striking in Mykolaivska and Donetska oblasts (Table 2). After adjustment for age, sex, and treatment phase (intensive or continuation), the rate ratio (RR) for the early phase of the war versus pre-war was 3.71 (95% confidence interval: 3.17–4.33), 1.95 (1.68–2.25), and 1.69 (1.52–1.87) in Mykolaivska and Donetska oblasts, Odeska oblast, and Lvivska and Zakarpatska oblasts, respectively. RRs remained elevated in the later phase of the war versus pre-war period. There was strong evidence for interaction by time period and region (*P* < 0.0001).

**Table 2.** Effect of time period, stratified by geographical area, on the percentage of treatment-days defined as ‘no information’ (unknown or manual doses added >7 days after treatment-day).

Area and time period	Total treatment-days	Total days with no information^**A**^ (%)	RR^**B**^ (95% CI)	RR^**C**^ (95% CI)
Mykolaivska/Donetska				
Before war	9,454	825 (8.7%)	1	1
26 February–29 April 2022	3,183	1,193 (37.5%)	3.71 (3.37, 4.61)	3.71 (3.17, 4.33)
30 April–28 October 2022	2,632	724 (27.5%)	3.06 (2.56, 3.65)	2.71 (2.27, 3.24)
Odeska (southern)				
Before war	12,662	1,053 (8.3%)	1	1
26 February–29 April 2022	5,751	951 (16.5%)	1.99 (1.73, 2.30)	1.95 (1.68, 2.25)
30 April–28 October 2022	11,900	1,930 (16.2%)	1.85 (1.64, 2.09)	1.72 (1.52, 1.93)
Lvivska/Zakarpatska (western)				
Before war	28,692	1,964 (6.8%)	1	1
26 February–29 April 2022	13,706	1,364 (10.0%)	1.66 (1.50, 1.84)	1.69 (1.52, 1.87)
30 April–28 October 2022	23,687	3,307 (14.0%)	2.00 (1.83, 2.18)	1.86 (1.70, 2.02)

RR = rate ratio (from negative binomial regression); CI = confidence interval.

A No information defined as a combination of i) unknown (no recording that the pillbox was opened and no additional information from the HCW) or ii) a manual dose added >7 days from the treatment-day.

B Adjusted for age and sex.

C Adjusted for age, sex, and treatment phase (intensive or continuation).

## DISCUSSION

Our analysis shows that the war has substantially affected patients’ and HCWs’ engagement with DATs. The war’s physical proximity had a more pronounced detrimental effect on engagement with the DATs, as seen in Mykolaivska and Donetska oblasts, which were the most affected by warfare.^18^ During the earlier stage of the war, the proportion of ‘no information’ treatment-days increased in all regions compared to pre-war, with the increase highest in Mykolaivska and Donetska oblasts. In the later phase of the war, increases from pre-war period in no information were still seen. While the increase of ‘no information’ days during the earlier stage of the war was expected, we did not expect that the change would be sustained in the later phase of the war.

Patients’ engagement with the DATs had declined since the beginning of the war, reflected by a decrease in digital treatment-days. Based on the intervention design, this would have resulted in an increase in the requirement for provider follow-up, placing greater burden on HCWs. Patient’s reduced engagement with the pillbox is likely a result of many war-related factors, including: an individual stress reaction to hostilities,^19^ unstable functioning of communication networks,^20^ and that TB patients, as with millions of other Ukrainians, were forced to leave their homes seeking a safer place within or outside of Ukraine,^21^ which could have resulted in problems with pillbox connectivity. These seem plausible, in particular, due to the striking reduction in patient and HCW engagement during the first 2 months of the war in Donetska and Mykolaivska oblasts, which were substantially more affected by the warfare than other regions. Having more detailed information on calls made by the HCW to patient would have been useful information to help confirm this hypothesis. An increase in no information following the start of the war in the southern and western regions versus pre-war, affected less by the war, could also be explained by high patient workload. Following the start of the war, people starting on TB treatment remained at similar levels compared with pre-war in these two regions. So despite DAT initiations reducing, HCWs who implemented the DAT intervention were still responsible for care of patients who were not enrolled in the study.

A key strength of our paper is providing evidence on the different trajectories of changes in HCW and patient engagement with the treatment and DATs over the course of the war and their variation across different geographies. This suggests that the effect of war is neither instantaneous nor uniform. This study takes a deeper look at the impact of the war on the mechanisms of TB care delivery, beyond the discrete epidemiological measurements like TB notifications or treatment outcomes, which are conventionally reported in regard to TB care under complex emergencies. The study has limitations. The first is the information on social, economic, psychological, infrastructural, patient-related, HCW-related, and health care system–related factors that could have led to the changes in the engagement with DATs. This limitation is somewhat inevitable due to the pragmatic nature of the ASCENT main trial. The analysis of the contextual factors extrinsic to the DATs intervention has been conducted within a qualitative sub-study on the HCWs’ acceptability and feasibility of DATs implementation, to be reported elsewhere. The second limitation is that the platform only recorded the HCWs’ interaction with the DAT engagement data, including manual confirmation of missed doses, whereas their further actions, envisaged by the differentiated care algorithm such as phone calls to the patients, were not automatically recorded. These data are important in understanding the HCWs’ engagement with the DATs and enabling us to explore the interaction between the HCWs and patients’ engagement with the DATs.^22^

## CONCLUSION

DATs allowed HCWs to monitor and provide support for patients who were migrating due to war, and indeed potentially improve treatment outcomes,^23^ though with all the described limitations. For the majority of patients in Ukraine, HCW in-facility DOT was absent for a significant period of time. Implementing the DAT intervention became substantially more complex when the setting faces a challenge, such as war. The war in Ukraine, already lasting for more than 36 months, is not the only war, nor the last one. The recent Tigray war in Ethiopia, an ongoing conflict in Yemen, and the civil war in Sudan make the need for innovative, resilient, and accessible interventions for TB treatment ever more urgent.^24^ Thus, if DATs are to provide a solution to supporting patient treatment, the interplay between the interventions and complex shifting contexts requires more attention from implementation scientists.

## Supplementary Material




