# Supplementary material for: The effects of war on digital adherence technology engagement for TB treatment in Ukraine
Source: IJTLD Open. 2026 Jan 9;3(1):24–30. doi: 10.5588/ijtldopen.25.0503 (PMC12826589; doi:10.5588/ijtldopen.25.0503)
Supplement: Supplementary file 1 [file ijtldopen25-0503_supplementarydata1.pdf]

**SUPPLEMENT to: Deyanova, McQuaid et al. The effects of war on digital adherence technology engagement for TB treatment in Ukraine**

## Narratives for trial settings and their war experiences

*Donetska oblast*

There is one rayon (cluster) in the trial from this oblast.

Part of Donetska oblast has been occupied by Russia since 2014. During the Russian invasion of Ukraine, by June 2022 around 55% of Donetska oblast came under the control of Russia.

The ASCENT intervention cluster is in Sloviansk district, situated within 60 km from Bakhmut - where a series of massive ground attacks and air shelling has been happening continuously since May 2022.

Since February 2022 there have been few days without shelling or destruction for Slovyansk residents who remained in the city. By June 2022 less than 24 000 inhabitants remained in Slovyansk, where 105 000 residents lived before the full-scale invasion[1].

TB-HCWs were evacuated in April 2022 but kept managing remotely those patients who remained in Ukraine.

*Mykolaivska oblast*

There are two rayons (clusters) in the trial from this oblast.

Mykolaivska oblast is located in southern Ukraine. The region had been directly affected by the Russian full-scale invasion. Initially, the region became an important outpost on Ukraine's southern frontline. The battle of Mykolaiv started on the night of 26 February 2022. Russian forces continuously and severely attacked Mykolaiv in February, March, and April.

Constant electricity outages and prolonged disruptions in the work of public transportation caused by continuous missile attacks severely limited patients' access to ASCENT intervention facilities.

Russian forces were repulsed from the city in April 2022 but managed to destroy the pipeline that brought fresh water to the city residents. People were forced to use and drink technical water, water from rivers and streams, and water donated by neighbouring regions for six months. Thousands of buildings across the Mykolaivska oblast remain battle-scarred because of constant shelling.

In early March 2022, the residents of Mykolaiv city began to flee their homes. Most TB healthcare workers involved in implementing the ASCENT study fled the city in April. In November 2022 approximately 240,000 residents remained in Mykolaiv, out of 480,000 who lived there before February 2022[2].

[1] https://www.unocha.org/attachments/c12ee306-e1f3-490d-92aa-a7adc0ea5f5d/Situation%20Report%20-%20Ukraine%20-%203%20Aug%202022.pdf

[2] https://www.icmpd.org/file/download/62233/file/Selected_Publications_2023-24_EN_web%2520%25281%2529.pdf

*Odeska oblast*

There are three rayons (clusters) in the trial from this oblast.

Located in southern Ukraine, along the northern coast of the Black Sea, the Odeska oblast is the region with the largest territory in Ukraine. It has eight seaports and remains one of the country's main logistic hubs. Odeska oblast has borders with Moldova and Romania.

The territory is divided by the estuary of the Dniester River, which lies as an obstacle to getting from the southern part of the region to the northern one.

Due to the large territory and the number of settlements dispersed across it, the distance between patients' homes and TB facilities can reach 80 km.

Since the full-scale invasion began, Russian forces have attacked the Odeska region many times by air and sea. During the first months of the war the missile strikes hit, among other targets, the main airport and seaport, and destroyed residential buildings, resulting in dozens of killed and wounded. However, there was no ground warfare in Odesa. The estimated number of IDPs in the Odesa region from other territories of Ukraine is about 150 000 people[3].

*Lvivska oblast*

There are two rayons (clusters) in the trial from this oblast.

Lvivska oblast is the largest region of western Ukraine, bordering Poland and having strong cultural and economic ties. Lviv city, the region's administrative center is only 90 km from the Poland border.

After the full-scale invasion began, there has not been air communication with Ukraine. Ground transportation is the only way for people to get from and to the country. And Lviv, due to its proximity to the EU, became the main passengers' logistics hub.

In 2023, Lvivska oblast's population reached 3 million people. Approximately 500,000 are internally displaced people, who fled the severe war actions in their home regions (mostly - in eastern and northern Ukraine) and moved to Lvivska oblast. Up to 600,000 IDPs have been staying in Lvivska oblast at the peak of displacement[4]. There were several episodes of shelling by Russian missiles, but it is considered a safer place to live.

*Zakarpatska oblast*

There are four rayons (clusters) in the trial from this oblast.

Geographically the westernmost region of Ukraine, Zakarpatska oblast has shared borders with Romania, Hungary, Slovakia, and Poland. For many years there used to be many cross-border labor migrants from the Zakarpatska oblast to the neighboring EU countries, which also caused a depopulation tendency in the region over several years consecutively.
More than half of the region's population lives in rural areas. Many settlements are situated in

---

[3] https://dtm.iom.int/reports/zvit-pro-vnutrishne-peremischennya-v-ukraini-opituvannya-zagalnogo-naselennya-raund-3-17

[4] https://www.unhcr.org/ua/en/news/unhcr-un-refugee-agency-and-lviv-regional-state-administration-solidify-ongoing-collaboration#:~:text=As%20one%20of%20the%20safer,at%20the%20peak%20of%20displacement

mountainous areas (the Carpathian Mountains). Given rare public transport, getting around is often challenging for people living there.

Due to its westernmost location, Russian aggression did not reach the Zakarpatska oblast directly. However, the size of the region's population grew by 30% due to the flow of internally displaced people coming to Zakarpatska oblast from other regions of Ukraine.[5]

Figure S1 – Map of Ukraine showing five ASCENT regions (oblasts) and their geographical proximity to the territories occupied by Russia. In the time period reflected in the analysis Mykolaivska and Donetska oblasts were the most affected by the warfare (ground fighting, missile attacks, and active hostilities); Odeska oblast was affected less (attacked by the Russian military forces from the sea and air, but there have been no ground military actions); Lvivska and Zakarpatska oblasts were least affected. This map is for illustrative purposes only; it should not be considered an exact and official representation of the situation (as of January 2024).

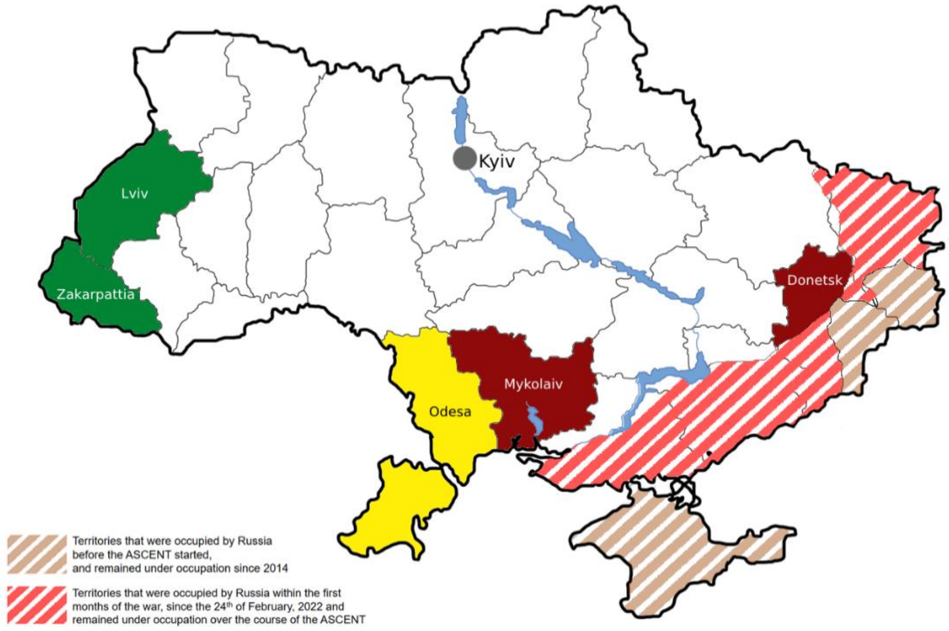


[5] https://www.undp.org/ukraine/press-releases/integration-hub-internally-displaced-ukrainians-opens-mukachevo
